# Supplementary material for: Extracting individual characteristics from population data reveals a negative social effect during honeybee defence
Source: PLoS Comput Biol. 2022 Sep 15;18(9):e1010305. doi: 10.1371/journal.pcbi.1010305 (PMC9477262; doi:10.1371/journal.pcbi.1010305)
Supplement: S1 Text — The supplementary material containing a detailed description of methods and tools employed in the methodological framework, including additional details on the obtained results and their reproducibility. (PDF) [file pcbi.1010305.s001.pdf]

Text S1 – Details of the methodology and results

Tatjana Petrov[2,3], Matej Hajnal[2,4], Julia Klein[2,3], David Šafránek[4], Morgane Nouvian[1,3,5]

[1] Department of Biology, University of Konstanz, Konstanz, Germany
[2] Department of Computer and Information Sciences, University of Konstanz, Konstanz, Germany
[3] Centre for the Advanced Study of Collective Behaviour, University of Konstanz, 78464, Konstanz, Germany
[4] Systems Biology Laboratory, Faculty of Informatics, Masaryk University Botanická 68a, 602 00 Brno, Czech Republic
[5] Zukunftskolleg, University of Konstanz, Konstanz, Germany

Abstract. In this supplementary material, we provide detailed information supporting the results described in the manuscript. First, we give formal definitions of the technical notions used in the manuscript. Second, we provide a detailed description of the model, experimental data and their encoding, generated rational functions, and individual computational methods employed in the performed analysis tasks. The description is extended with two additional computation methods that have been used to additionally support the obtained results (parameter space sampling and refinement).
Reproducibility of the results is supported with all necessary input files and software codes. All input files (the model, analysed properties, data, and the results) are described in this supplementary text and are available on Zenodo `https://zenodo.org/record/6600766#.YpbF13VBw9E`. The software artefacts are described in the file README.md located with the software in the publicly accessible GitHub repository (`https://github.com/xhajnal/DiPS`).

1 Preliminaries

Definition 1 (MC). A Markov Chain is a tuple $\mathcal{M} = (S, P, \iota_{init}, AP, L)$ over a countable, nonempty set of states S. The transition probability function $P : S \times S \to [0, 1]$ is such that $\sum_{s' \in S} P(s, s') = 1$ for all $s \in S$, the initial distribution $\iota_{init} : S \to [0, 1]$ is such that $\sum_{s \in S} \iota_{init}(s) = 1$. Set of atomic propositions is denoted by AP, and a state-labelling function $L : S \to 2^{AP}$ assigns a set of labels to each state in S.

Given an MC $\mathcal{M} = (S, P, \iota_{init}, AP, L)$, the probability space is assigned in the standard way, i.e. for any $l \geq 0$, the prefix set of traces $\sigma = (s_0, s_1, \ldots, s_l) \in S^l$

⋆ Authors acknowledgements are declared in the respective section of the main manuscript.

is assigned the probability measure $\mathsf{P}_{\mathcal{M}}(\sigma) = \iota_{init}(s_0)\prod_{i=0}^{l-1} P(s_i, s_{i+1})$. The property of reaching a BSCC in a Markov Chain can be written in a temporal logic PCTL (Probabilistic Computational Tree Logic) [8]. We here consider a fragment of PCTL properties. These are defined over the traces for MC $\mathcal{M}$ in a standard way, by state formulae induced by the grammar $\Phi ::=$ true $\mid a \mid \Phi \mid \Phi_1 \wedge \Phi_2 \mid \Phi_1 \vee \Phi_2 \mid P_J(\phi)$, where $a \in AP$, ϕ is a path formula, and $J \subseteq [0,1]$ is an interval, and path formulae $\phi ::= \Phi_1 \mathsf{U} \Phi_2$, where Φ_1, Φ_2 are state formulas. We will write $\mathsf{P}(\mathcal{M} \models \phi) = \sum_{s \in S} \iota_{init}(s)\mathsf{P}_{\mathcal{M}}(s \models \phi)$ to denote the probability of satisfaction of PCTL property ϕ in the MC $\mathcal{M}$.

When the transition probabilities are not known, but rather are rational functions of some parameters from the parameter set $\mathcal{V}$, each over domain $[0,1]$, we work with a parametric Markov Chain (pMC). We here restrict our attention to the case when the transition probabilities are multivariate rational functions over the variables $\mathcal{V}$, which we will denote by $\mathsf{Pol}_{\mathcal{V}}$. [6]

Definition 2 (pMC). A Parametric Markov Chain (pMC) is a tuple $M_{\mathcal{V}} = (S, P_{\mathcal{V}}, \iota_{init}, AP, L, \mathcal{V})$, where $P_{\mathcal{V}} : S \times S \to \mathsf{Pol}_{\mathcal{V}}$ defines the probability transition matrix, and for each evaluation of parameters $\theta \in [0,1]^{|\mathcal{V}|}$ induces a well-defined MC $M(\theta) = (S, P_\theta, \iota_{init}, A, L)$, where P_θ denotes the instantiation of the expression $P_{\mathcal{V}}$, for parameter evaluations given by a vector θ. Consequently, for any $\theta \in [0,1]^{|\mathcal{V}|}$, for all $s \in S$, $\sum_{s' \in S} P_\theta(s, s') = 1$.

Definition 3 (BSCC). Given $\mathcal{M}$, a subset T of S is called strongly connected if for each pair (s,t) of states in T there exists a path $s_0 s_1 ... s_n$ such that $s_i \in T$ for $0 \leq i \leq n$, $s_0 = s$, $s_n = t$, and $P(s_i, s_{i+1}) > 0$ for all $i = 0, \ldots, n-1$. A strongly connected component (SCC) of $\mathcal{M}$ denotes a strongly connected set of states such that no proper superset of T is strongly connected. A bottom SCC (BSCC) of $\mathcal{M}$ is an SCC T from which no state outside T is reachable, i.e. for each state $t \in T$ it holds that $P(t, T) = 1$.

We denote the steady-state distribution of an MC by $\mu : S \to [0,1]$. Since almost surely any finite Markov Chain eventually reaches a BSCC and visits all its states infinitely often, the steady-state distributes the probability mass among its BSCCs, i.e.$\sum_{T \in BSCC(\mathcal{M})} \sum_{s \in T} \mu_s = 1$ [2].

We will employ Bayesian approaches to estimate parameters agreeing with data.

Definition 4 (Bayes theorem). Let $\pi(\theta)$ denote the prior distribution over parameter(s), $P(D_{obs}|\theta)$ the likelihood of data observations under given parameters, and $\int_\theta P(D_{obs}|\theta)\pi(\theta)d\theta$ the marginal distribution of data observation. Then, the posterior distribution $\pi(\theta|D_{obs})$ evaluates to $\pi(\theta|D_{obs}) = \frac{P(D_{obs}|\theta)\pi(\theta)}{\int_\theta P(D_{obs}|\theta)\pi(\theta)d\theta}$.

[6] In general, the reachability probabilities for a pMC can be expressed by rational functions; in case studies shown in this paper, polynomials will suffice.

2 Experimental Data

2.1 Groups of 10 bees

The input data list, D, visualised in Figure A in S1 Text as barplot, is:

$$[0.2391304348, 0.152173913, 0.2065217391, 0.1195652174, 0.04347826087, 0.1086956522, 0.08695652174, 0.02173913043, 0.02173913043, 0, 0]$$

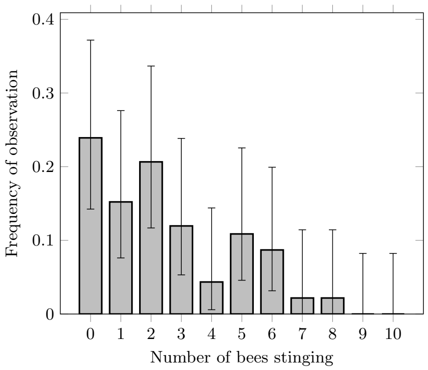

Figure A: Experimental data with Agresti-Coull confidence intervals (using Dunn's correction for simultaneous confidence intervals for multiple parameters) and 90% confidence level. Frequencies of the number of stinging bees $(22, 14, 19, 11, 4, 10, 8, 2, 2, 0, 0)$ resulting from 92 repeated experiments.

It is available as text file within the Zenodo repository at `https://zenodo.org/record/6600766#.YpbF13VBw9E`. As the data points, $D = (\hat{x}_1, \ldots, \hat{x}_k)$, are sheer estimates, to address uncertainty of the data one can create confidence intervals around respective data point $\hat{x}_i$. From the data we have obtained the intervals, I, (shown as error bars in Figure A in S1 Text) using 92 samples and overall confidence level of 90% for all eleven parameters. We calculated corrected confidence level per parameter using Dunn's correction as follows: $corrected\_confidence\_level = 100 - (10/11) = 99.0909090909$. To address the poor approximation when applied to Bernoulli trials with p close to 0 or 1, we computed confidence intervals using Aggresti-Coull method [1]:

$$\begin{aligned}
&(0.142404365423760, 0.371789667663575),\\
&(0.0761601111095509, 0.276098599673562),\\
&(0.116847754788942, 0.336620532434309),\\
&(0.0531606878280316, 0.238372277090998),\\
&(0.00583076995249482, 0.144008787950341),\\
&(0.0457848450804105, 0.225506204550591),
\end{aligned}$$

$$(0.0315535645967751, 0.199253654458171),$$
$$(0.0, 0.114342459711623),$$
$$(0.0, 0.114342459711623),$$
$$(0.0, 0.0822898761868151),$$
$$(0.0, 0.0822898761868151)$$

These confidence intervals are further used in one of the supported methods (space sampling and refinement).

2.2 Full datasets

The complete sets of the three experiments described in Table 1 in the manuscript are visualised in Figure B in S1 Text. At each row, the raw data of the particular dataset are shown.

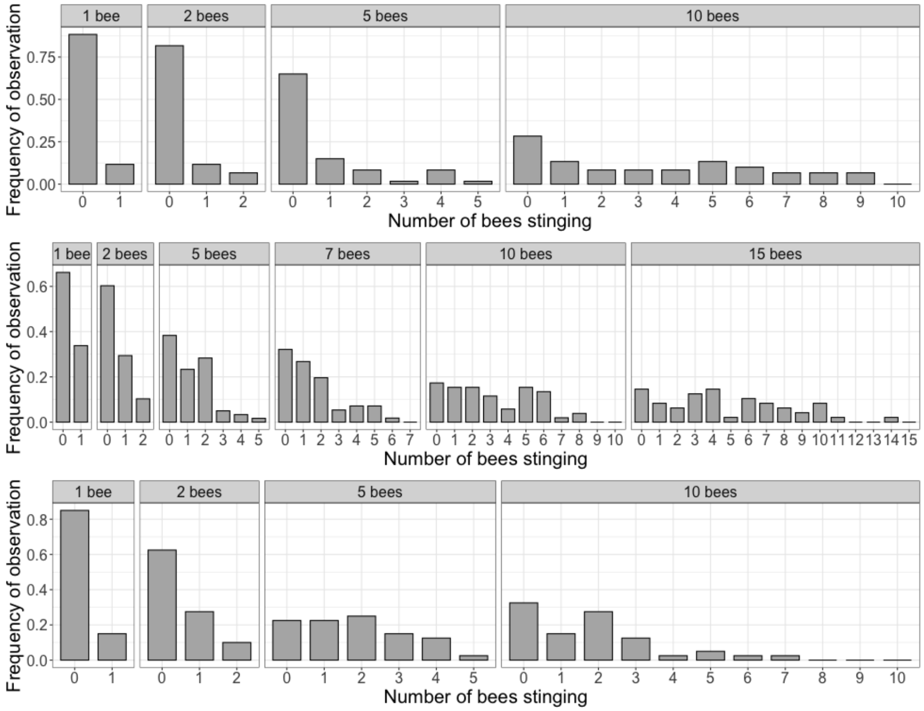

Figure B: Raw experimental data of dataset 1, dataset 2, and dataset 3. Frequency at which a given number of stings is observed at the end of the test duration, for each group size. The sample sizes (number of groups tested) are $N_1 = [60, 60, 60, 60]$, $N_2 = [68, 68, 60, 56, 52, 48]$ and $N_3 = [40, 40, 40, 40]$ ordered from the smallest group size (1 bee) to the largest (10 or 15 bees depending on the dataset).

3 Model

In Figure D in S1 Text we show an extension of the model from Figure C(C) in S1 Text to three bees. It is worth noting that here we do not display the full model (ten bees) due to its size. The full model is available at `https://zenodo.org/record/6600766#.YpbF13VBw9E`. To represent the model, we utilise the abstract PRISM modelling language in `.pm` format (the states and transition probabilities are explained in Figure D in S1 Text. To obtain a model for a given population size we created a Python script, which makes a part of the tool package. The script automatically generates the state space of the model.

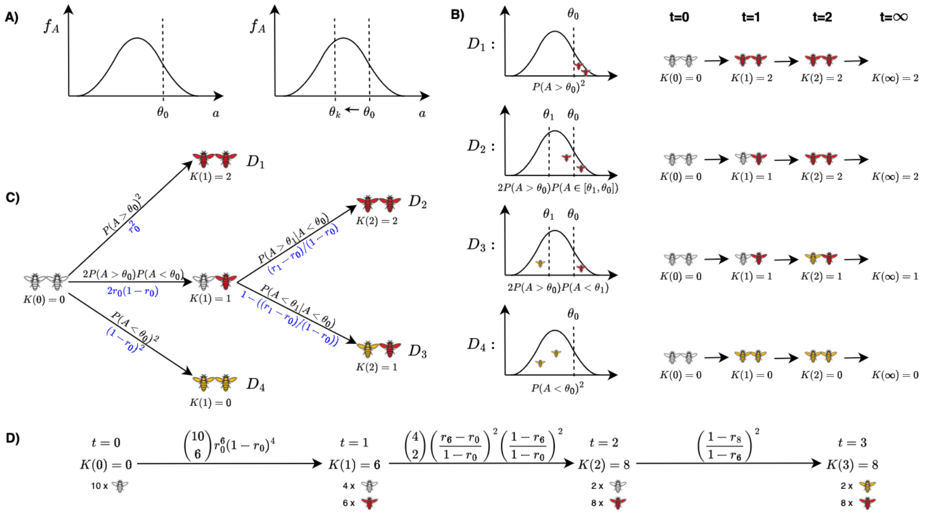

Figure C: Modelling stinging response for $n = 2$. A) Example probability density function of aggressiveness. B) Four different situations for a group of two bees. Simulation of different stinging behaviour across time, for four different initial aggressiveness situations. C) A Markov chain model of four different stinging scenarios for a group of two bees. D) An example trace of a Markov chain model generated for $n = 10$.

Non-decreasing parameter values We propose an assumption/constraint on parameters values. In the transitions of the model (see Figure D in S1 Text), the term encoding the probability of a bee stinging when i amount of pheromone given the bee did not sting at k amount of pheromone equals:

$$\frac{r_i - r_k}{1 - r_k}. \tag{1}$$

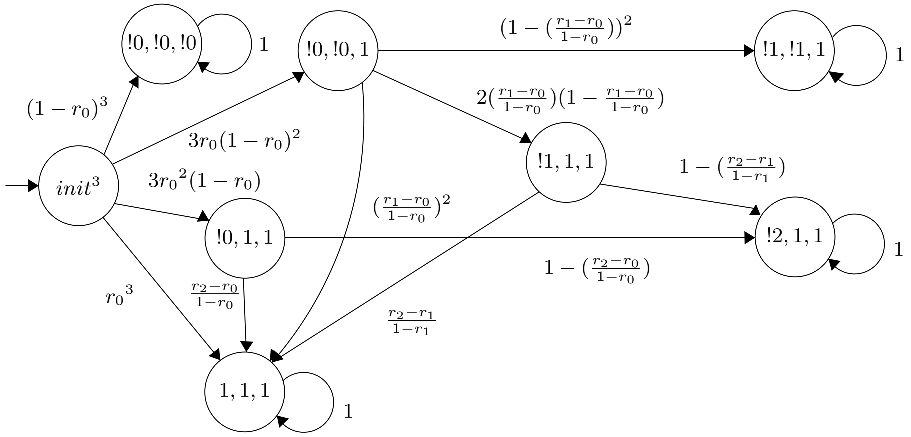

Figure D: Model of three bees as a parametrised Markov Chain. In each state, $(a_1, \ldots, a_n, b)$, a vector depicting the state of the population is shown, where each bee agent, $a_1, \ldots, a_n$, tracks its state - *init* before stimulus, 1 stinging, and $!i$ not stinging when i amount of pheromone is present, i.e. $A < \theta_i$. Flag b indicates whether there is no bee which could sting, hence whether we have reached the steady-state (not shown in Figure) – this helps us to query the model using temporal logic. Transitions, updates of bee states whether to sting or not, are parametrised by the probability of stinging when i amount of pheromone is present - r_i. When multiple bees are updating their state, we consider this as an independent decision and hence multiply respective terms. Terms such as $\frac{r_k - r_i}{1 - r_i}$ interpret the conditional probability $P(A > \theta_k \mid A < \theta_i)$.

Our model assumes that the probability r_i is not smaller than r_k, i.e. $r_i \geq r_k$, otherwise the term describing conditional probability would not be well-defined (since it could take a negative value). To avoid the possibly ill-defined transition probabilities, the value should be 0 in case $r_i < r_k$. Since neither PRISM nor Storm is capable of working with if-then clause nor $max(0, \frac{r_i - r_k}{1 - r_k})$, we constraint the parameter values of the resulting rational functions. We have adapted DiPS for this purpose with the implementation visible on the branch `non-decreasing`. Another option is to introduce fresh parameters for each of these terms. However, this would increase the number of parameters quadratically as there are up to $\frac{n \cdot (n-1)}{2}$ parameter pairs to be substituted.

4 Methodology

4.1 Data Encoding

To estimate valid parametrisations of the model which agree with the data, a link between the events observed in the experiments and the events in the model has to be drawn. In our case, the number of bees stinging at the end

of an experimental trial represents reaching the respective BSCC in the model. Probabilities to reach each of the BSCCs can be calculated manually using all the paths leading to the BSCC and common prefixes reflected, e.g. using the inclusion-exclusion technique, to compute the value or the symbolic expression over model parameters. With the increasing size of the model, this task becomes tedious and even infeasible for human. To resolve this, if the data are formalised in form of temporal logic, *model checking*, an automated verification method, can be used instead. We use a commonly used Probabilistic Computational Tree Logic (PCTL) for pMCs. Each temporal property is querying the probability to reach a single BSCC:

$$P_{=?}[F(a_0 = 0)\&(a_1 = 0)\&(a_2 = 0)\&\ldots\&(a_9 = 0)\&(b = 1)], \qquad (2)$$

where the probabilistic operator $P_{=?}$ enumerates the probability of the formula given in the square brackets. It consists of the future operator, F, denoting the eventual reachability of a state satisfying the enclosed formula. The atomic propositions forming the enclosed formula express the state properties. The variable a_i has the range $\{0, 1\}$ where $a_i = 1$ (resp. $a_i = 0$) encodes the fact ith bee is stinging (resp. not stinging). The proposition $b = 1$ encodes the "steady-state" property of the state (the state forms a trivial BSCC). Details of the state variables are shown in Figure D in S1 Text. After the properties are created, one can exactly compute the probabilities of satisfaction using parameter synthesis [10]. In that case, the result is not a single value but an expression over model parameters having the form of a *rational function*.

4.2 Rational Functions

Model checking is a decision problem answering whether a model satisfies a given property. In the probabilistic setting, using quantitative properties, properties with prefix $P_{=?}$, the result is not True or False, but the probability of satisfaction. Moreover, having the model parametrised, the probabilities of satisfying given property depend on the values of the model parameters. Hence, *parametric model checking* returns a symbolic expression over parameters; in the case of pMC models in the form of *rational functions*.

In our case study, each rational function estimates the probability of reaching respective BSCC in the model encoding number of stinging bees. In particular, we obtain 11 rational functions, encoding events of 0, 1,..., 10 bees stinging, with 11 model parameters, $(r_0, r_1, \ldots, r_n)$. Let us denote the rational functions as $F = (f_0, f_1, \ldots, f_n)$. From this point we will refer to probability of seeing i stinging bees as f_i. To obtain the rational functions DiPS leverages existing tools Storm [5] or PRISM [11]. After that, we factorise the rational functions to facilitate parameter estimation.

Without the rational functions the probability of satisfaction of a PCTL formula, probabilities of reaching respective BSCC in our case, one needs to run the pMC chain in order to the estimate the probability instead of simply evaluating the rational functions. As this has to be done in each parameter point to be

analysed, the estimation becomes imprecise and/or expensive. Moreover, global and exact methods can be applied with knowledge of the rational functions.

We have obtained the rational functions using DiPS, which calls PRISM to run parametric model checking of the model and a temporal property of reachability of respective BSCC one-by-one. For instance $P(K(\infty) = 5) = f_5 = -252r_0(r_5 - 1)^5(r_0^4 - 5r_0^3r_4 - 10r_0^2r_3^2 + 20r_0^2r_3r_4 - 10r_0r_2^3 + 30r_0r_2^2r_4 + 30r_0r_2r_3^2 - 60r_0r_2r_3r_4 - 5r_1^4 + 20r_1^3r_4 + 30r_1^2r_3^2 - 60r_1^2r_3r_4 + 20r_1r_2^3 - 60r_1r_2^2r_4 - 60r_1r_2r_3^2 + 120r_1r_2r_3r_4)$. One can see there are 16 different paths to obtain 5 stinging bees as 16 different monomials (in the last bracket). We do not show all the rational functions due to their length.

Linear and sigmoidal models were created by substituting r_i with right-hand side of respective equations in each rational function (result of parametric model checking of the agnostic model).

4.3 Non-triviality of the Likelihood Function

Here we demonstrate that even with sigmoidal model one can not draw the probabilities of reaching respective BSCC from a simple distribution. We show this by using a single sigmoidal parametrisation: $r_0, K_m, V_{max}, n =$ [0.1341, 0.8401, 0.2329, 3], which resolves in $r_0, r_1, \ldots, r_n =$ [0.1341, 0.1961, 0.2261, 0.2308, 0.232, 0.2324, 0.2326, 0.2327, 0.2328, 0.2328] with rational functions $f_0, f_1, \ldots, f_n =$ [0.237, 0.188, 0.2005, 0.1992, 0.1173, 0.0444, 0.0114, 0.002, 0.0002, 0, 0], while it has two peeks - at the first and the third function.

4.4 Implemented Computational Methods

The results have been primarily obtained using the DiPS tool (Data-informed Parameter Synthesiser) which is available open source at `https://github.com/xhajnal/DiPS`. It is worth noting that besides the two methods presented in the manuscript, DiPS adds two additional methods for parameter estimation of DTMCs – *Space Sampling* and *Space Refinement*. Here we describe all four methods and their adaptations used in the manuscript:

- **Optimisation** finds a single point in the parameter space with the least L2 distance wrt. data,
- **Metropolis-Hastings** identifies a distribution over all possible parametrisations based on their relative likelihood wrt. data using Bayesian inference,
- **Space sampling** marks single points in the parameter space satisfying (green) or not (red) the constraints [4] obtained from data,
- **Space refinement** marks entire regions (hyper-rectangles) in the parameter space within which the constraints are always satisfied (green), or not (red), using either a SMT solver [4, 7, 9, 10] or interval arithmetic, and

Each method provides its pros and cons, which can be mainly depicted as a trade-off between computational demand and the type of information they provide. In the rest of this section we introduce respective methods with its input structures. For complete description please follow DiPS tutorial at `https://github.com/xhajnal/DiPS/blob/master/tutorial.pdf`.

Optimisation *Optimisation* searches parameter space $\Theta \subset \mathcal{R}^m$ and returns a single parametrization $\hat{\theta} \in \mathcal{R}^m$ which minimises given objective function, in our case L2 distance - square root of the sum of squares of distances between the k rational functions, F, st. $f_i(\theta) \in \mathcal{R}$, and k data points, D, st. $\hat{x}_i \in \mathcal{R}$:

$$\hat{\theta} := \underset{\theta \in \Theta}{argmin} \sum_{i \in \{1,\ldots,k\}} (f_i(\theta) - \hat{x}_i)^2$$

With the knowledge of the rational functions, optimisation is a classical solution for this problem, however as the result is a single point, it can be far from the true value - as an example consider a rational function $p \cdot q$ and experimental observation of 0.2. This can be described as a function $p \cdot q = 0.2$ with a hyperbolic curve as the result. The regression will return point [0.44, 0.44] but absolutely disregards continuous range of other parameters values resulting with rational function value with the same distance. For this reason we have implemented other methods which deal with this problem.

Metropolis-Hastings *Metropolis-Hastings* belongs to a group of Markov Chain Monte Carlo (MCMC) methods. For a given number of iterations t it walks in parameter space $\Theta \subset \mathcal{R}^m$, producing a sequence of points $\theta_0, \theta_1, \ldots, \theta_t$. At each point it decides whether to accept or to reject a new point. In particular, the acceptance rule compares the probability of the new sample point θ', $P(\theta'|D)$, with the probability of the current sample point θ, $P(\theta|D)$. Using Bayesian rule,

$$P(\theta|D) = \frac{P(D|\theta) \cdot P(\theta)}{P(D)}.$$

As we do not consider any prior knowledge of the distribution of parameters values, the uniform distribution is used. Moreover, as the probability of the data is equal in both points, the likelihood of the samples, $P(D|\theta')$, are compared:

$$\frac{P(\theta'|D)}{P(\theta|D)} = \frac{P(D|\theta') \cdot P(\theta')}{P(D)} \cdot \frac{P(D)}{P(D|\theta) \cdot P(\theta)} = \frac{P(D|\theta')}{P(D|\theta)}$$

The new point, θ', is accepted if the fraction is greater than one. In the case the fraction is smaller than one, to avoid local optima, Metropolis-Hastings accepts the new point with a small probability.
If the new point, θ', is accepted, we move to that point, $\theta_i = \theta'_{i-1}$, otherwise we keep the current point, $\theta_i = \theta_{i-1}$. After the walk is done, the set of accepted points serves as a prediction of the distribution of viable parameter values - posterior distribution.

We adapted the Optimisation and Metropolis-Hastings to search in only non-decreasing parameter subspace as discussed in Section 3.

Note that a different setting (not default), especially the value of deviation of the transition function, may have significant impact on the result, e.g., the number of accepted points.

Constraints In general, any set of (in)equalities over the set of parameters, $\mathcal{V}$, can serve the purpose of constraining the parameter space. In our case, we use the confidence intervals, I, computed from the data points, $\hat{x}$. As a result, each of the rational function, f_i, is within the respective interval, I_i, creating this conjunction:

$$\bigwedge_{k=0}^{n} (f_i \in I_i).$$

Space Sampling In the first step of the method, a uniform grid of a given size in each dimension of parameter space is created. Consequently, constraints are checked in each of these points and if all of the constraints are satisfied the parameter point is marked as satisfying, sat for short, and marked as unsatisfying, unsat, otherwise. The meaning of unsat reflects the situation when at least one of the constraints is not satisfied.

Space Refinement Similarly as in the space sampling, refinement checks the satisfaction of the constraints. However, to obtain a global result instead of discontinuous parameter points, regions are checked instead. In the initial step, the whole parameter space is verified to satisfy the constraints. If all of the points within the space satisfy the constraints, the space is marked as safe. If all the points within the space do not satisfy the constraints, the space is marked as unsafe. If neither of the two holds, there is a sat and an unsat point within the space, the space is split into two and the procedure is called recursively. This process continues until the specified coverage is reached (a given threshold of the fraction of the safe and unsafe subspace).

To check whether the given space is safe or unsafe we use satisfiability modulo theory (SMT) solvers z3 [3] and dreal [6], or interval arithmetic of scipy [12]. SMT solver is an extension of Boolean satisfiability problem (SAT), decision problem whether there exists satisfying evaluation of a given Boolean formula. SMT extends SAT with theories expressed in first-order logic and more structures, in our case we utilise (in)equalities over real numbers.

To learn more about the tool and learn step-by-step usage with an example, please read the `tutorial.pdf` available at GitHub. Thanks to DiPS, the entire workflow is fully reproducible. Both input files – the model, properties, and the data – as well as the results described in the following sections of the supporting material (Optimisation Results, Metropolis-Hastings Results, Model Selection, and Sampling-Based Local Sensitivity Analysis) are available at Zenodo (`https://zenodo.org/record/6600766#.YpbF13VBw9E`).

5 Results

5.1 Optimisation Results

The results, optimised parametrisation and respective rational functions in the point, provided in this section are visualised in Figure E in S1 Text. Due to stochastic nature of optimisation algorithms one can obtain slightly different parametrisation, therefore we round the result values to 4 decimals.

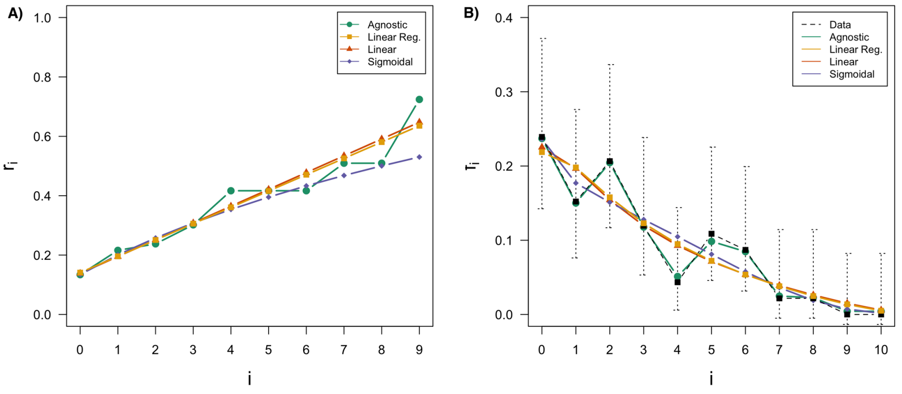

Figure E: A) Single point estimation (parametrisation minimising the L2 distance between the rational functions and data). B) For comparison, rational function values (coloured lines), data (dashed line), and 90% confidence intervals computed from data points (black error bars).

Agnostic Model Optimisation results in parameter point, $[r_0, \ldots, r_9] = [0.1341,$ 0.2159, 0.2379, 0.3019, 0.4167, 0.4167, 0.4167, 0.5094, 0.5094, 0.7241]. In this point, the rational functions, $[f_1, \ldots, f_{10}]$, evaluate to: [0.2371, 0.1501, 0.2045, 0.1178, 0.0512, 0.0985, 0.0847, 0.0248, 0.0228, 0.0043, 0.0043] with the distance to data equal to 0.0153.

Linear Model Optimisation results in parameter point, $[r_0, delta]$: [0.0566, 0.1385]. In this point, the rational functions, $[f_1, \ldots, f_{10}]$, evaluate to: [0.2253, 0.1965, 0.1543, 0.1197, 0.0927, 0.0713, 0.0538, 0.0391, 0.0262, 0.0151, 0.0059] with the distance to data equal to 0.1019.

Sigmoidal Model Optimisation results in parameter point, K_m, V_{max}, n, r_0: [20.0, 1.3827, 0.9604, 0.1344]. In this point, the rational functions, $[f_1, \ldots, f_{10}]$, evaluate to: [0.2364, 0.177, 0.1511, 0.1279, 0.1048, 0.0811, 0.0577, 0.0362, 0.0189, 0.0073, 0.0016] with the distance to data equal to 0.098.

5.2 Metropolis-Hastings Results

The results obtained with the Metropolis-Hastingsmethod can be almost exactly reproduced, assuming that the numbers of iterations as specified below are followed. Detailed description of the settings and results is shown in Table A in S1 Text.

Agnostic model Result posterior distribution as a result of 10 million iterations is shown in Figure F(A) in S1 Text. We have run the Metropolis-Hastings for twice many iterations to check the convergence of the method at 10 million iterations. Thanks to the convergence, a replica with the same or higher number of iterations should result in almost the same figure.

Linear model We have adapted Metropolis-Hastings algorithm for the linear model such that it walks in viable parameter space, using constraint $r_0+9\Delta \leq 1$. Otherwise would r_9 evaluate to a value above 1. Result posterior distribution as a result of 30 million iterations is shown in Fig 6B.

Sigmoidal model Result posterior distribution as a result of 358,287 iterations is shown in Figure F(C) in S1 Text. This particular instance of Metropolis-Hastings was kept running for similar time. However, since the rational functions of the sigmoidal model are only partially factorised the procedure went through significantly fewer points.

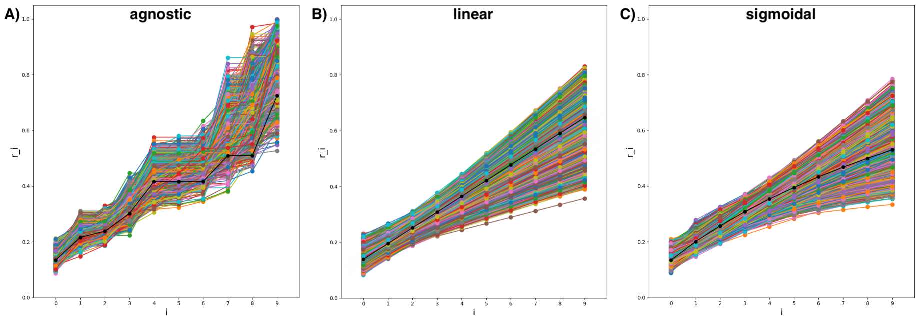

Figure F: Metropolis-Hastings results of the agnostic (A), linear (B), and sigmoidal (C) model: set of accepted points. Each accepted point shown as a line with values of respective parameter point. Burn-in period selected as 25%. We run the agnostic model for twice many iterations to check the convergence of the method. The black line shows the respective optimised point.

	# iterations	# accepted points	burn-in period	initial parameter	computational time	# parameters
Agnostic	10,000,000	2,249	25%	[0.1, ..., 0.1]	15.67h	10
Linear	30,000,000	546,934	25%	[0.5, 0.5]	12.56h	2
Sigmoidal	358,287	19,841	25%	[50, 5, 5, 0.5]	96h	4

Table A: Individual settings and corresponding results achieved with the Metropolis-Hastings method for agnostic, linear, and sigmoidal model (shown in particular rows). The columns display the following information respectively: the total number of points explored; the number of accepted points; the proportion of accepted points which were trimmed out from the beginning; the initial parametrisation; the computation time (in hours); and the number of dimensions of the explored parameter space (the number of explored parameters).

5.3 Model Selection

We computed the AIC scores for the linear and the sigmoidal model to choose the best model that fits our data. First, the AIC is based on the rational function values f_i:
$AIC_f(\text{lin}) = 11\ \log(0.0103837400504974/11) + 2 \cdot 2 = -72.6195036836891$
$AIC_f(\text{sig}) = 11\ \log(0.0094159680459984/11) + 2 \cdot 4 = -69.6956793295523$
Next, we compute the AIC scores based on the parameter values r_i (RSS compared to agnostic model):
$AIC_r(\text{lin}) = 10\ \log(0.020280751758762/10) + 2 \cdot 2 = -58.0066812496777$
$AIC_r(\text{sig}) = 10\ \log(0.044737975830065/10) + 2 \cdot 4 = -46.0951765989556$

Our previous results suggest that the sensitivity of the parameters r_i to the model output decreases for increasing i, and the uncertainty increases. Therefore, we weigh the fit of the parametrization based on the ranges of accepted points (variance) we obtain from the Metropolis-Hastings algorithm (Figure F(A) in S1 Text). The weights are computed using the min-max normalization of the ranges $w = [w_0, ..., w_9]$: $1 - \frac{w_i - \min(w)}{\max(w) - \min(w)}$. We then multiply the weights with the residuals of the respective model to get normalized residuals to the true parameter values. We get the following AIC scores based on normalized residuals:

$AIC_{r\_n}(\text{lin}) = 10\ \log(0.00318550133041276/10) + 2 \cdot 2 = -76.5173069175542$
$AIC_{r\_n}(\text{sig}) = 10\ \log(0.00306976526194241/10) + 2 \cdot 4 = -72.8873927520845$

We observe that the AIC score is lower for the linear model in all three variants, hence we conclude that the linear model is preferred over the sigmoidal model. However, the AIC only evaluates the relative quality of the model compared to other models, not the absolute quality. Therefore, we need to validate the absolute quality of the linear model, which usually includes to check the model's residuals and predictions.
Figure G(a) in S1 Text shows the residuals of the linear model on the vertical axis, and the independent variable (parameters) on the horizontal axis. We see that the residuals are randomly dispersed around the horizontal axis, what indicates that the linear regression model is appropriate for the data and a good

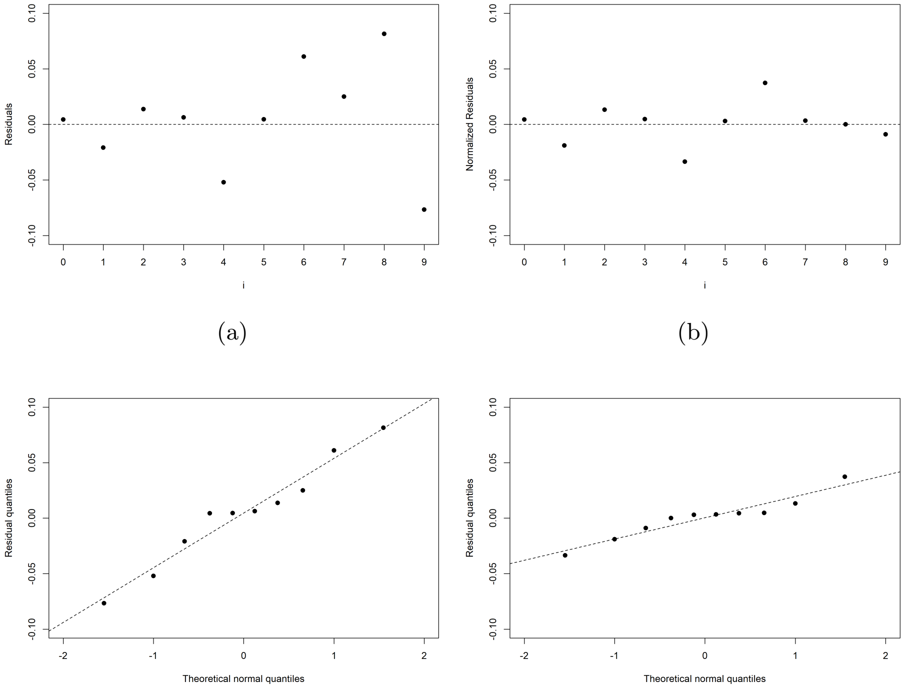

(a) (b) (c) (d)

Figure G: Residual plots of the parameter values obtained by the linear model. a) Residuals of linear model (compared to the agnostic model). b) Normalized residuals. c) Q-Q plot to check that residuals come from a normal distribution. d) Q-Q plot of normalized residuals.

fit. Figure G(b) in S1 Text confirms this result for normalized residuals.
Figure G(c) in S1 Text and Figure G(d) in S1 Text are Q-Q plots, showing quantiles of the data versus quantiles of the normal distribution. We see that the points follow a straight line, what indicates that the residuals are drawn from a normal distribution. This supports our assumption that the linear model is a good fit of the data.

The next step in model validation is to test the model's predictions. We compute the coefficient of determination, R^2, as a measure of the predictive power of the linear model based on the parameter values r_i:
$R^2(\text{lin}) = 1 - \frac{0.020280751758762}{0.26899700837561} = 0.924606032307828$
Again, we repeat the computation for normalized residuals:
$R_n^2(\text{lin}) = 1 - \frac{0.00318550133041276}{0.117114003801542} = 0.972799996353888$
High R^2 values confirm that the linear regression model fits the data well, because the predictions approximate the real data points. More specifically, the results tell us that 92.42% (resp. 97.28%) of the variation in the observations is explained by the model.

5.4 Sampling-Based Local Sensitivity Analysis

To evaluate the uncertainty related to each of the parameter values inferred by optimisation, we have implemented a local sensitivity method based on sampling. In more detail, the method samples parameters one by one while fixing all but one single parameter. A single parameter value of the sampled point is used to recompute the distance. Sampled points which obtained a lower distance than the given parametrisation are visualised with blue colour and those with the distance below five quarters of the distance of the given parametrisations are shown in red. We checked the agnostic model (Figure H in S1 Text), linear model (Figure I in S1 Text), and sigmoidal model (Figure J in S1 Text). In the visualisations, with blue colour highlights the intervals of sampled parameter values with lower distance, while the red colour highlights the intervals which contain sampled point with the distance lower that five quarters of the original distance. There are 10 000 points (with uniform distribution) sampled for each of the parameters.

In the results achieved with all models, one can see that parameters with lower indices are much more sensitive. Intuitively, this can be explained by the fact that those parameters have impact on more rational functions involved, and especially on the rational functions with lower indices.

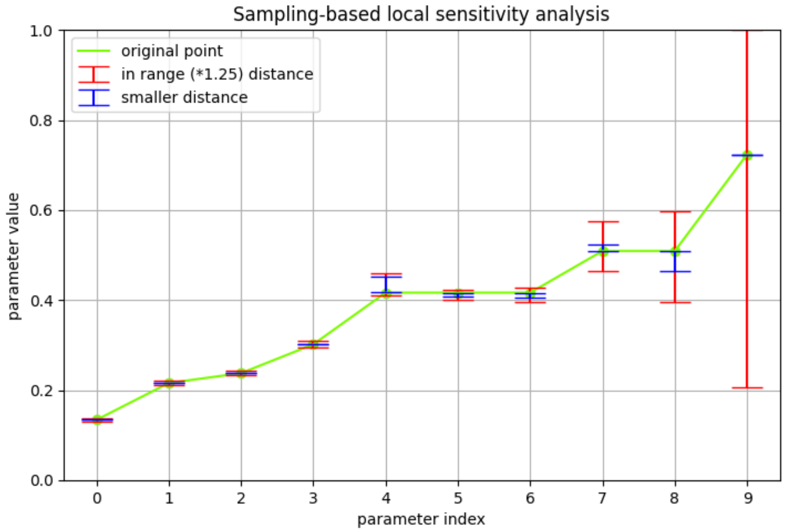

Figure H: Sampling-based local sensitivity analysis from optimised point of the agnostic model.

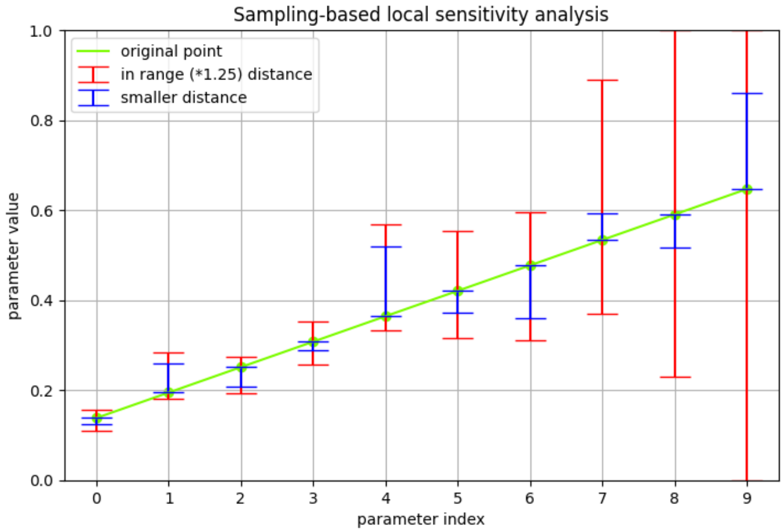

Figure I: Sampling-based local sensitivity analysis from optimised point of the linear model.

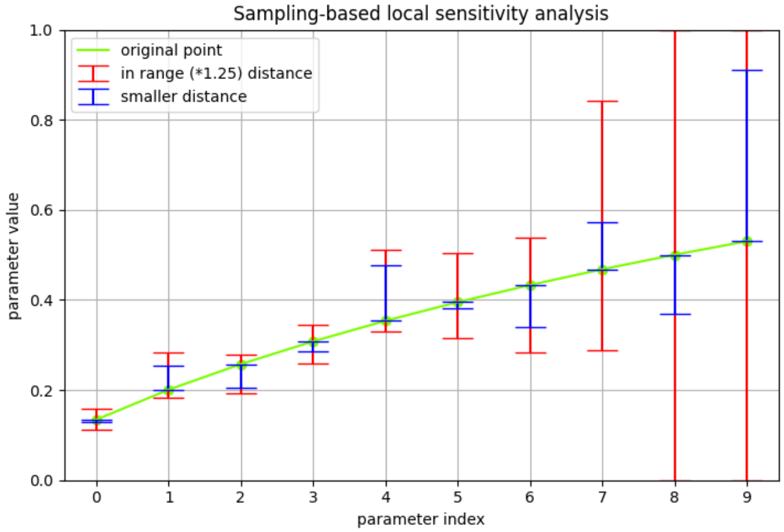

Figure J: Sampling-based local sensitivity analysis from optimised point of the sigmoidal model.

5.5 Application to different group sizes

To study the effect of group size on stinging likelihood, we applied our methods on our three experimental datasets. Figure K in S1 Text presents the results obtained with the agnostic method (points, and linear regression on those points in solid lines), optimisation of the linear model (dash-dotted lines) and optimisation of the 4-parameter sigmoidal model (dashed lines), for each group size (depicted with different colours). As expected, all methods produced similar results, broadly revealing that individual bees became less likely to sting (r_i) at any given units of alarm pheromone released (i). Note that the difference between the linear model and the linear regression on agnostic points is caused by the fact that the linear model better accounts for sampling uncertainty of experimental data points. In order to determine which of these models was the best fit for our data, we then computed the AIC scores for all of them (Table B in S1 Text). When comparing to the experimental data (Rat. functions), we found that the linear model performed best (bold values) most often (6 cases out of 11, compared to 2 and 3 cases for the linear regression on agnostic points and sigmoidal model, respectively). Hence, we used the linear model for our detailed analysis of the biological results (see main text).

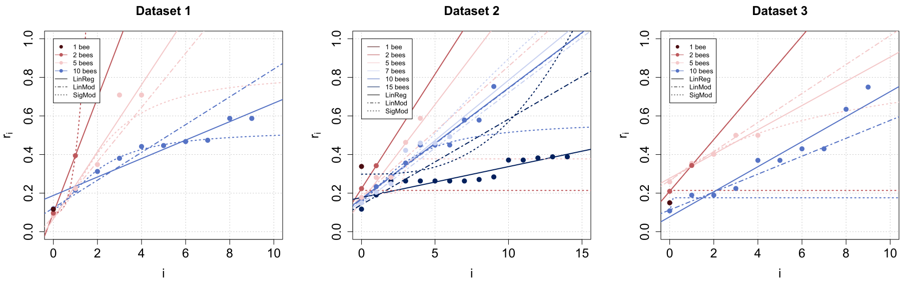

Figure K: Results of dataset 1, dataset 2, and dataset 3. Points: non-decreasing agnostic method. Lines obtained with linear regression on agnostic model (solid), linear model optimisation (dash-dotted) and sigmoidal model optimisation (dashed). The colours correspond to individual group sizes.

References

1. Alan Agresti and Brent A Coull. Approximate is better than “exact” for interval estimation of binomial proportions. *The American Statistician*, 52(2):119–126, 1998.
2. Christel Baier and Joost-Pieter Katoen. *Principles of model checking*. MIT press, 2008.
3. Leonardo Mendonça de Moura and Nikolaj Bjørner. Z3: An efficient SMT solver. In *TACAS'08*, volume 4963 of *LNCS*, pages 337–340. Springer, 2008.

		Dataset 2				
		n=2	n=5	n=7	n=10	n=15
Rat. functions	Lin. reg.	**-77.04429**	-22.96957	-44.2734	-62.01428	-80.95236
	Lin. mod.	-66.60726	**-24.68541**	**-46.27075**	**-62.06285**	**-96.99849**
	Sig. mod.	-3.87599	-16.02526	-43.54569	-61.34852	-77.1656
Parameters	Lin. reg.	**-143.88720**	**-28.37871**	**-40.77465**	**-61.72313**	**-101.0043**
	Lin. mod.	-69.65053	-22.07014	-37.66669	-61.65702	-43.62552
	Sig. mod.	-1.591831	-12.85816	-33.40121	-40.28781	-32.5585

		Dataset 1			Dataset 3		
		n=2	n=5	n=10	n=2	n=5	n=10
Rat. functions	Lin. reg.	-69.9176	-27.28591	-50.8566	**-72.1645**	-32.02892	-49.56424
	Lin. mod.	-66.28502	**-32.45099**	-62.51448	-66.78661	**-33.51561**	-56.7964
	Sig. mod.	**-70.41448**	-28.78085	**-72.56585**	-3.384777	-30.88411	**-65.36278**
Parameters	Lin. reg.	**-148.2568**	**-23.07141**	**-60.11463**	**-144.7488**	**-33.9039**	**-54.46849**
	Lin. mod.	-71.06902	-19.40156	-43.80958	-72.29325	-30.60723	-44.29791
	Sig. mod.	-66.63377	-15.82454	-54.70355	-1.556828	-32.84847	-17.95251

Table B: AIC scores of all three datasets to evaluate the fits of the implemented models: linear regression on agnostic points, linear model and sigmoidal model. Scores for rational functions were computed by comparing the function evaluations to the experimental data, and for parameters by comparing the parameter values to the points obtained by optimisation with the agnostic model. The numbers in bold report the best results for individual cases.

4. Christian Dehnert, Sebastian Junges, Nils Jansen, Florian Corzilius, Matthias Volk, Harold Bruintjes, Joost-Pieter Katoen, and Erika Ábrahám. *PROPhESY: A PRObabilistic ParamEter SYnthesis Tool*, pages 214–231. Springer, 2015.
5. Christian Dehnert, Sebastian Junges, Joost-Pieter Katoen, and Matthias Volk. A STORM is coming: A modern probabilistic model checker. In *Computer Aided Verification*, pages 592–600. Springer, 2017.
6. Sicun Gao, Soonho Kong, and Edmund M. Clarke. dReal: An SMT solver for nonlinear theories over the reals. In *CADE-24*, volume 7898 of *LNCS*, pages 208–214. Springer, 2013.
7. Ernst Moritz Hahn, Tingting Han, and Lijun Zhang. Synthesis for PCTL in Parametric Markov Decision Processes. In *Nasa formal methods symposium*, pages 146–161. Springer, 2011.
8. Hans Hansson and Bengt Jonsson. A logic for reasoning about time and reliability. *Formal Aspects of Computing*, 6(5):512–535, Sep 1994.
9. Sebastian Junges, Erika Ábrahám, Christian Hensel, Nils Jansen, Joost-Pieter Katoen, Tim Quatmann, and Matthias Volk. Parameter synthesis for markov models. *CoRR*, abs/1903.07993, 2019.
10. Joost-Pieter Katoen. The probabilistic model checking landscape. In *Proceedings of the 31st Annual ACM/IEEE Symposium on Logic in Computer Science*, pages 31–45. ACM, 2016.
11. Marta Kwiatkowska, Gethin Norman, and David Parker. Prism 4.0: Verification of probabilistic real-time systems. In *International conference on computer aided verification*, pages 585–591. Springer, 2011.
12. Stefano Taschini. Interval arithmetic: python implementation and applications. In *Proc 7th Python Sci Conf (ScyPy 2008)*, 2008.
