# Supplementary material for: Extracting individual characteristics from population data reveals a negative social effect during honeybee defence
Source: PLoS Comput Biol. 2022 Sep 15;18(9):e1010305. doi: 10.1371/journal.pcbi.1010305 (PMC9477262; doi:10.1371/journal.pcbi.1010305)
Supplement: Attachment — Submitted filename: Bees_paper_revision_PlosCB-review_response.pdf [file pcbi.1010305.s002.pdf]

Review Response

We would like to thank all the reviewers for valuable comments. Below we give our reactions on the individual comments and describe the relevant changes in the manuscript. The original reviewers comments are typeset in italic.

Reviewer #1

In this manuscript, the authors develop and use a new technique, using a combination of exact and statistical methods, to infer individual behaviors from only the group response. They apply this technique to study recruitment of a defense behavior in a group of honeybees. In this experiment, a fake predator was presented to a group of 10 bees, and the number of bees that stung the decoy was counted. The authors then inferred the probability that a bee initiates a stinging behavior, given a certain number of bees have already stung the decoy.

I think that the main strength of this work is in the inference technique that is developed and deployed. The general problem of inferring individual behaviors only from measurements of a collective outcome is very common in the field of collective behavior, and this methodology should prove to be very useful for many researchers. The authors have describe the methodology clearly by building up the reader's intuition by first describing a single bee, then two bees, then generalizing to an arbitrary number of bees.

I think this manuscript's contribution to our understanding of honeybees is somewhat more limited. They do extend our understanding of defense recruitment in honeybees, which may then be compared to other recruitment behaviors in honeybees, or in other species. It would have been interesting, however, to have tested other group sizes, or a larger group size. Most interesting collective behaviors occur at group sizes much larger than $N = 10$, so gaining some insight at those larger groups would have been useful.

On balance, I think the theoretical contributions outweigh the limited experimental contributions and would support this paper to be published in this journal.

We thank Reviewer 1 for their positive assessment of our work. Following these comments and those of the other reviewers, we have now added a section comparing different group sizes (we had this data but originally thought that it would be too much for this manuscript). We show that there is a negative social effect on honeybee recruitment, such that the individual likelihood to sting at a given alarm pheromone concentration decreases as group size increases (L439-475). As a consequence, our contribution in terms of understanding honeybees is now much more developed. We agree that other interesting things may happen at even larger group sizes, but both our experimental assay and the analytical methods presented here are still limited in their scalability (the main bottleneck being the generation of an exponential number of rational functions describing our model), so this will have to remain as our next challenge for now.

Reviewer #2

This study proposes a modeling approach to investigate the collective attack behavior of honey bee colonies during nest defense. The argument is that it can be difficult to scale single individual behavior to collective, group-level responses. Unfortunately, I do not have the expertise to comment on the modeling approach itself, but I believe the investigators have identified an important problem, and one that could benefit from a modeling approach. I hope my comments improve the impact of this manuscript.

Given the extensive simplifying assumptions of the model (acknowledged by the authors themselves in the Discussion), I feel the value of the model and its impact are overstated. The authors state "Thanks to the new model and tools presented here, we'll now be able to expand the study to larger group sizes, which was previously impossible." While this may be true in the simplified arena context, does this study get us any closer to understanding what is going on inside a beehive during a predator attack?

For example, the authors highlight the importance of social context in predicting the escalation of the anti-predator response, but they do not really wrestle with what is already understood about the complexity of this response, and they do not fully justify why their approach retains value despite ignoring this complexity. For example, contrary to the model assumptions, there are a variety of studies suggesting negative, not positive social feedback in response to alarm pheromone and other defensive cues, both at the colony level and in lab-based assays (e.g., Kastberger et al. 2009, Rittschof 2017, papers with first author Hagai Shpigler). It seems like a lot of modern studies on honey bee aggression are ignored in this study. Given this (and other assumptions listed below), the model is overly simplistic. I understand that it may be a first step towards understanding this phenomenon (as mentioned in the Discussion), but the impacts of the current model seem overstated.

We thank Reviewer 2 for pointing out what was actually the starting idea of our work, although we agree that it was not apparent in the previous version of our manuscript. As you can now appreciate from the data we added (L439-475), which is comparing group sizes and indeed revealing a negative social feedback, we are very aware of this possibility. Most importantly, our model does reproduce the effect of negative feedback though it is not explicitly included in the model. Thus, we believe that our model is indeed a useful tool to advance our understanding of honeybees. In addition to the result section mentioned above, we also added a comprehensive discussion on this topic (L531-558). While we appreciate the value of the studies cited, we also have reserves about the results they report. Kastberger at al. 2009 did not provide a moving target for the bees to attack, which is essential to trigger flying and stinging responses (as demonstrated by Free 1961, Wager and Breed 2000 and their own study, in which pheromone presentation alone failed to elicit a response). Rittschof 2017 is indeed a good example of negative social effect, but in the context of defence against non-nestmates which is quite different from the defence against vertebrates considered here (we clarified this point). As for papers from Shpigler (2017, 2019), they mainly show that individual bees remain consistent in their aggressive behaviour, again in the context of defence against non-nestmates. We are not sure how that fits into the argument so we didn't include them, please let us know if you were referring to another paper that we missed.

The authors list many critical caveats and assumptions of their model and the ways in which it fails to capture real-world biology. As a result, an informed reader is left wondering about the benefits of the model at all. To counteract this impression, the authors could do more to explain why, despite the simplicity, this modeling approach is meaningful. This should occur throughout the manuscript, not just in the Discussion. The approach would come across better if it better justified the simplifications, and perhaps gave specific examples of the ways that these could be addressed in future studies.

We hope that the concrete application of our model included now is the best proof of its benefits. We also tried to make our point clearer in the discussion (L409-503 and L536-542).

More detailed comments related to model simplifying assumptions that could be addressed:

How might the results of this assay in which the predator does not leave or escalate the attack track the real-world dynamics of predator response? Similarly, what are the implications of ignoring the possibility that stings may build up slowly versus quickly?

It is true that a confrontation is a two-sided process, in which the predator's actions also play a role. We feel, however, that adding this level of complexity is outside of our reach at the moment, and outside of the focus of the current manuscript. Regarding the slow vs fast build up of stings, note that our model is not based on real time: one step corresponds to one sting, regardless of whether it took 10s or 2min for the bee to sting. The main assumption is therefore that the alarm pheromone does not significantly degrade during our assay. We have done a few PID measures inside the arena which seem to support this idea. Nevertheless, considering the real time dynamics of the system including factors such as alarm pheromone degradation would indeed be extremely interesting — but extremely challenging. Again, we have to leave that for future studies at this stage.

The nature of the intruder context influences whether bees show positive or negative social feedback for attack – this issue, i.e., the type of predator considered, is not clear.

We consider the case of a vertebrate, typically much larger than bees. This has been clarified L35+45.

L44 most defensive behaviors are low level behaviors (your data seem to support this as stinging is relatively infrequent) — please address the implications of only measuring sting response.

Stinging may appear infrequent because (to our dismay), bee colonies in New-Zealand and Germany are strongly selected for their gentleness. More to your point though, stinging responses are the most efficient in deterring large predators and also the costliest in terms of worker depletion, since they nearly always result in the death of the bee. They are thus the most likely to be tightly regulated by social context, which was our main interest in this study. In addition, low level behaviours are much more difficult to quantify, and we do not know if/how they would influence other bees.

L84 The size of the population impacts information transfer because odor signals diffuse over physical space. How can the results with the current model be extrapolated to "any" population size, as the model ignores this component? This seems like an overstatement.

We moderated this statement (L100). However, population size should not play a strong role in odour diffusion within the limited space of our arena.

I think it would help in the Methods to contextualize the lab study with the real-world predator attack. For example, the time frame chosen was 10 min because few bees sting after this time period in the lab assay, but how does that relate to a real-world predator attack?

We would love to be able to do that, unfortunately we have never been able to find data characterizing vertebrate attacks on bee colonies. If you do know of some please contact M.N., she will be thrilled.

L158 – while the model assumptions correspond to the arena assay, they do not correspond to real-world conditions, which is the fundamental challenge to understanding collective behavior at a hive scale.

These are some extremely significant assumptions that are contradicted by real-world conditions:

-Pheromone doesn't degrade over time

-Spatial homogeneity is assumed, which is not realistic for a bee hive.

-Bees equally influence each other, which is a simplification esp given the negative feedback that can occur

-If multiple bees react simultaneously (L168), it seems like temporal dynamics are particularly important to consider.

More should be done to justify (or just simply explain) the approach, particularly in the Methods.

We agree and this is why we tried to openly acknowledge these limitations as much as possible. But we are still convinced that our approach gets us closer to understanding collective behaviour also at the scale of the colony. Reducing the complexity of a system by transferring it into controlled laboratory conditions is and has always been among the first steps of any scientific endeavour. We hope that the added section comparing group sizes now provides a good example of how our model can help in dissecting the factors influencing an individual's decision to sting, one at a time... until one day we can fit them all together and bring this theory back into the field, that would be the dream! Rather than expanding our very long methods, we tried to make this point clearer in the discussion (L510-518 and L536-542).

L172 – individuals are known to have intrinsically different thresholds, especially across patrilines, which occur within any naturally mated colony – here you assume they are all the same. You revisit this issue in the Discussion and say that the model DOES account for different response thresholds. How or why is unclear to me – I'm not sure how to reconcile the Discussion with L172.

We are not sure if we understand this comment (and if 172 is the correct line number?). We do not assume that all bees are the same, their stinging thresholds vary (if their stinging thresholds were all the same, we would see an all or nothing response). This is written in the methods (section "stinging behaviour of an individual bee", L193-198) and can be seen in the Appendix on Fig. 2 (on which we take the distribution of thresholds to be normal, but this is only for representation purposes. Our model does not actually assume a specific shape for this distribution).

Other detailed comments:

L14 Unclear of purpose of this sentence.

In this paragraph we review studies showing that individuals can behave differently depending on their social context, and this is another example of this. We do not understand why this is unclear.

L16 what is meant by "mechanistic understanding"?

We clarified our meaning (L17-18).

L30 honey bees gather nectar not honey (except in extreme cases like robbing)

They gather nectar but they store honey, which is what we meant here. The sentence has been corrected (L30-31).

L35 guards can emit alarm pheromone while standing at the entrance

Corrected (L35-37).

L103 contraction

This sentence was removed during revisions.

L184 – you refer to a colony specific threshold but it is not clear to me how this is defined.

This is an important mistake, and we thank Reviewer 2 for catching it. θ_0 is not a "colony-specific threshold", but rather a "predator-specific threshold" representing how "scary" the predator needs to be to trigger stinging without alarm pheromone. This has now been corrected (L198-199). Note also that θ_0 is very similar to r_0 in the sense that they both refer to the pheromone level 0, even though they are not strictly equivalent quantities (one is stinging probability, the other is aggressiveness threshold).

L336 – is "wrt" "with respect to", or another acronym? Please write out.

Yes it is. This was corrected throughout the manuscript.

Reviewer #3

This is an exciting topic, that many beekeepers, bee researchers, and bee enthusiasts meet: once you get stung once, there's a good chance you'll get stung again due to recruitment. By combining experiments and modeling, the authors show that the collective decision making is based of individuals sensing the alarm pheromone concentration. Overall, the paper is written clearly, the methods are sound, but I do have some major concerns about the interpretation of the results:

1. The analysis of the experiments is somewhat superficial, i.e., the authors only count the final number of stingers at the end of the experiment. Wouldn't measuring the temporal value of the number of stingers provide a stronger model validation? At the moment the model validation is entirely dependent on the data presented in Fig. 2.

Measuring the timing of each stings requires advanced tracking techniques, because bees can jump on and off the dummy multiple times, and we often observe that they take the stinging posture even after they already lost their stinger. And manual scoring gets really complicated when you have more than 5 bees, as the attacks are usually fast and close in time. We have a large dataset of videos that we hope will provide this type of information. However, our aim with this manuscript is to provide methods that are amenable to easily quantifiable group measurements, as we explain in the introduction (L20-29). Hence, we think that this is actually a strength in our approach rather than a weakness.

2. If the model cannot be better validated (point 2), the authors should at least provide some testable predictions, allowing for model validation in future experiments (e.g., predict what would happen for groups of different sizes, as the authors mention in the introduction and abstract).

We have addressed this point by adding a section in which we actually compare group sizes (we had this data but initially thought that it would be too much for a single paper). We hope that this makes the validity and value of our model obvious (L439-475).

3. Collection of bees: it is not clear if each experiment consisted of 10 bees from the same colony, or if they were mixed from different colony. This is an important detail, because bees from different colonies could exhibit defense response towards each other, hence altering the social dynamics of the group.

Within a group, the bees were always from the same colony, so they never displayed aggression towards each other. This was clarified in the methods (L136).
